# Methylation of drug resistance‐related genes in chemotherapy‐sensitive Epstein–Barr virus‐associated gastric cancer

**DOI:** 10.1002/2211-5463.12765

**Published:** 2019-12-03

**Authors:** Hirofumi Ohmura, Mamoru Ito, Keita Uchino, Chihiro Okada, Shigeki Tanishima, Yuichi Yamada, Seiya Momosaki, Masato Komoda, Miyuki Kuwayama, Kyoko Yamaguchi, Yuta Okumura, Michitaka Nakano, Kenji Tsuchihashi, Taichi Isobe, Hiroshi Ariyama, Hitoshi Kusaba, Yoshinao Oda, Koichi Akashi, Eishi Baba

**Affiliations:** ^1^ Department of Medicine and Biosystemic Science Kyushu University Graduate School of Medical Sciences Fukuoka Japan; ^2^ Department of Clinical Oncology NTT Medical Center Tokyo Japan; ^3^ Engineering Section Biomedical Informatics Development Department Kansai Division Mitsubishi Space Software Hyogo Japan; ^4^ Department of Anatomic Pathology Graduate School of Medical Sciences Kyushu University Fukuoka Japan; ^5^ Department of Pathology National Hospital Organization Kyushu Medical Center Fukuoka Japan; ^6^ Department of Gastrointestinal and Medical Oncology National Hospital Organization Kyushu Cancer Center Fukuoka Japan; ^7^ Department of Internal Medicine Munakata Medical Association Hospital Fukuoka Japan; ^8^ Department of Internal Medicine Kyushu University Beppu Hospital Oita Japan; ^9^ Department of Oncology and Social Medicine Kyushu University Graduate School of Medical Sciences Fukuoka Japan

**Keywords:** chemotherapy, DNA methylation, drug susceptibility, EBV, Epstein‐Barr virus‐associated gastric cancer, gastric cancer

## Abstract

Epstein–Barr virus (EBV)‐associated gastric cancer (GC) is associated with a high degree of DNA methylation. However, the association between chemotherapy susceptibility and tumor DNA methylation in advanced diseases remains unclear. The comprehensive DNA methylation status of GC cells obtained from an advanced EBV‐associated GC (EBVGC) case, in which complete response to S‐1 plus cisplatin chemotherapy was achieved, was analyzed using a DNA methylation microarray. We compared DNA methylation of GC cells with public data and identified genes with higher methylation in EBVGC cell lines than in normal gastric cells, and genes in which methylation was increased by EBV. Of these genes, *ABCG2, AHNAK2, BCL2, FZD1,* and *TP73* are associated with published evidence for resistance to 5‐fluorouracil and cisplatin. Silencing of these genes may be associated with hypersensitivity to chemotherapy.

## Graphical abstract

Here, we report an analysis of comprehensive DNA methylation of gastric cancer (GC) cells obtained from an advanced Epstein–Barr virus (EBV)‐associated GC case, which showed complete response to standard chemotherapy, using the latest DNA methylation microarray platform (Illumina Infinium MethylationEPIC BeadChip). We compared DNA methylation of GC cells with public data and identified genes in which methylation was increased by EBV.

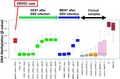

**Abbreviations**
EBER‐ISH: EBV‐encoded small ribonucleic acid‐in situ hybridization
EBV: Epstein–Barr virus
EBVGC: EBV‐associated GC
GC: gastric cancer
MSI: microsatellite instability
TCGA: The Cancer Genome Atlas

Gastric cancer (GC) is one of the most common malignancies and is the third leading cause of cancer‐related death in the world 1. For patients with advanced and recurrent cases, the main treatment is platinum‐ and fluoropyrimidine‐based systemic chemotherapy. However, the median survival time has been reported to be around 12 months and the prognosis remains poor. Therefore, the development of novel therapies targeting the genomic characteristics of patients’ cancer cells is urgently required 2. Epstein–Barr virus (EBV) infects ~ 90% of all adults, and, although most cases show latent infection, infectious mononucleosis sometimes occurs. Moreover, EBV associates with several malignancies such as Burkitt’s lymphoma, Hodgkin’s lymphoma, nasopharyngeal carcinoma, and gastric carcinoma. EBV‐associated GC (EBVGC) shows the unique histological features of lymphoepithelioma‐like carcinoma. EBVGC is diagnosed based on EBV‐encoded small ribonucleic acid‐in situ hybridization (EBER‐ISH) positivity in carcinoma cells, which suggests the infection by EBV. EBVGC accounts for around 10% of all GC and tends to occur in young males 3. The Cancer Genome Atlas (TCGA) consortium recently classified GC into four molecular subtypes: EBV, microsatellite instability (MSI), genomically stable, and chromosomal instability 4. EBVGC is thus a distinct molecular subtype and is characterized by *PIK3CA* mutation, overexpression of PD‐L1/PD‐L2, and hypermethylation of DNA. DNA methylation of promoter regions in certain cells causes gene silencing and can cause tumorigenesis 3. In EBVGC, promoter methylation has been associated with the silencing of several tumor suppressor genes (*CDK2AP2, CDKN2A, PTEN, APC*) and epithelial‐to‐mesenchymal transition (EMT)‐related genes (*CDH1, THBS1*), which suggests a relationship with oncogenesis and proliferation 3, 5. Although EBVGC is generally associated with a better prognosis than EBV‐negative GC 6, the potential relationship between DNA methylation and chemosensitivity in this context has not yet been elucidated. Moreover, global DNA methylation analyses in EBVGC have not been widely reported. Thus, the methylation status of individual cases and the methylation pattern of each gene have been difficult to analyze comparatively using existing databases. In the present study, we conducted a comprehensive DNA methylation analysis using the latest microarray platform (the Illumina Infinium MethylationEPIC BeadChip, or EPIC) on a highly chemosensitive EBVGC case and established a method to perform comparative analysis with existing databases from different cell populations. Using this approach, we found that the genes associated with chemosensitivity were highly DNA methylated in the EBVGC case, suggesting that gene silencing plays an important role in drug resistance in this disease.

## Material and methods

### Case presentation

A 59‐year‐old man with a tumor in the upper gastric body and cervical and celiac multiple lymphadenopathy was admitted to a local hospital, where a biopsy of the cervical lymph node was carried out. Histopathologically, the tumor tissue was negative for blood cell markers (CD3, CD30, CD45RO, PAX5, CD138) and positive for an epithelial marker (cytokeratin AE1/AE3) and EBER‐ISH, consistent with an epithelial malignancy. Upper gastrointestinal endoscopy revealed a submucosal tumor‐like lesion, 4–5 cm in diameter, at the posterior wall of the upper gastric body. Histopathological examination of the endoscopic biopsy from this lesion revealed it to be a poorly to moderately differentiated adenocarcinoma. HER2 was negative by immunohistochemistry (IHC), and EBER‐ISH was positive. The patient was thus diagnosed with EBVGC (TXN3M1, cStage IV). PD‐L1 was positive in both the primary and metastatic lesions by IHC, with 15% PD‐L1‐positive cells recorded in each lesion. The patient was treated with standard chemotherapy for advanced gastric carcinoma, using S‐1 and cisplatin. S‐1 was orally administered at 80 mg·m^−2^·day^−1^ on days 1–21, and cisplatin was intravenously administered at 60 mg·m^−2^·day^−1^ on day 8, followed by a 2‐week rest. We then repeated this 5‐week therapeutic cycle. A remarkable decrease in the sizes of the primary lesion and metastatic lymph nodes was observed during the course of therapy. After two cycles of therapy, tumor shrinkage was 54%, defined as a partial response according to the Response Evaluation Criteria in Solid Tumors (RECIST version 1.1), and after eight cycles, a complete response had been achieved. S‐1 alone was continued after the eighth cycle. After 14 cycles, the endoscopic needle biopsy from the primary lesion showed no proof of malignancy. Fluorodeoxyglucose (FDG)‐positron emission tomography also showed no abnormal uptake of FDG. The patient is alive without relapse, 36 months after initiation of treatment.

### DNA methylation analysis

In this EBVGC case, tumor‐genomic DNA was extracted from a formalin‐fixed, paraffin‐embedded (FFPE) biopsy specimen of the cervical lymph node and assessed using the Infinium HD FFPE QC assay protocol (Illumina, Inc., San Diego, CA, USA). DNA samples were bisulfite‐converted with the EZ DNA Methylation Kit (Zymo Research, Irvine, CA, USA) and DNA methylation was analyzed using the Infinium MethylationEPIC BeadChip (Illumina, Inc.). Data analysis was performed with the Illumina GenomeStudio Methylation Module (Illumina, Inc.). The intensity of DNA methylation at individual CpG sites was described as β‐values, which is calculated as the ratio of M/ (M + U), where M is the methylated signal intensity and U is the unmethylated signal intensity. β‐Values range from 0 in the case of a completely unmethylated CpG site to 1 in a completely methylated CpG site 7. The study protocol was approved by the ethics review board of Kyushu Medical Center (Fukuoka, Japan) in May 2015, and written informed consent was obtained from the patient. All procedures followed were in accordance with the ethical standards of the responsible committee on human experimentation (institutional and national) and with the Helsinki Declaration of 1964 and later versions. The methylation data have been deposited in NCBI's Gene Expression Omnibus 8 and are accessible through GEO Series accession number http://www.ncbi.nlm.nih.gov/geo/query/acc.cgi?acc=GSE132406. A public methylation dataset (accession number http://www.ncbi.nlm.nih.gov/geo/query/acc.cgi?acc=GSE89269, NCBI, Gene Expression Omnibus) 9 was acquired to compare with the EBVGC case. The dataset consisted of seven methylation analyses from different cell types, as follows: (a) nine samples of the normal human gastric epithelial cell line (GES1) experimentally infected with EBV over time up to day 28 (GES1_rEBV_days), and cells prior to experimental EBV infection (GES1_WT; accession numbers for array‐based data, GSM2363415–GSM2363423); (b) five samples of the hypomethylated human GC cell line MKN7 experimentally infected with EBV over time up to day 28 (MK7_rEBV+_days), and cells prior to EBV infection (MKN7_WT; GSM2363424–GSM2363428); (c) the human EBVGC cell line SNU‐719 (GSM2633598); (d) the human EBVGC xenograft KT (GSM2633598); (e) hypomethylated clinical GC specimens not associated with EBV (cancer_LowGC_1 and cancer_LowGC_2; GSM2363431, GSM2363432); (f) hypermethylated clinical GC specimens not associated with EBV (cancer_HighGC_1 and cancer_HighGC_2; GSM2363433, GSM2363434); and (g) two samples of normal gastric mucosal cells (control_NFGM_1 and control_NFGM_2; GSM2363429, GSM2363430). Since the platform used in the downloaded dataset was the Infinium Human Methylation450K BeadChip (Illumina, Inc.), which is the previous version of the Infinium MethylationEPIC BeadChip, comparison with the EBVGC case in the present study was made using probes common to the two platforms. It has been reported that at the time of upgrading from 450K to EPIC, more than 90% of the probes were carried over and thus common between the platforms 10. DNA methylation was determined to be positive for probes with β values > 0.4 and negative for those with β values < 0.2. Promotor regions were defined as 1500 base pairs upstream of the transcriptional start site. For comparison of data between samples, Wilcoxon signed‐rank testing, hierarchical clustering (Ward’s method and Euclidean distance), and heatmap analyses were performed using the statistical analysis software r 11. We next searched methylation microarray data of EBVGC patients from TCGA (project name; TCGA‐STAD) in the National Cancer Institute's database, Clinical Data Commons (https://portal.gdc.cancer.gov/). Twenty‐five of Infinium Human Methylation450K BeadChip data from TCGA were available and they were combined with the data of our EBVGC case in the same way explained above, and the methylation patterns were compared with heatmap and clustering analysis. The case ID of EBVGC cases from TCGA are as follows: TCGA‐B7‐5818‐01A, TCGA‐CD‐5801‐01A, TCGA‐CG‐5722‐01A, TCGA‐D7‐5577‐01A, TCGA‐BR‐6455‐01A, TCGA‐BR‐6706‐01A, TCGA‐BR‐6707‐01A, TCGA‐BR‐7196‐01A, TCGA‐FP‐7916‐01A, TCGA‐FP‐7998‐01A, TCGA‐BR‐7958‐01A, TCGA‐BR‐8285‐01A, TCGA‐BR‐8366‐01A, TCGA‐D7‐8570‐01A, TCGA‐D7‐8573‐01A, TCGA‐BR‐8381‐01A, TCGA‐BR‐8589‐01A, TCGA‐BR‐8676‐01A, TCGA‐BR‐8686‐01A, TCGA‐HU‐8608‐01A, TCGA‐HU‐A4G6‐01A, TCGA‐BR‐A4J4‐01A, TCGA‐D7‐A4YX‐01A, TCGA‐HU‐A4G2‐01A, TCGA‐HU‐A4H0‐01A.

### Immunohistochemistry

PD‐L1 staining in the EBVGC case was performed using FFPE samples of the primary lesion and the metastatic lesion at the cervical lymph node using an anti‐PD‐L1 antibody (Clone 28‐8; Abcam, Cambridge, UK). FFPE samples were sliced to 3 μm, and slides boiled to activate the antigen. Primary antibody was then applied, and secondary antibodies subsequently applied for detection. The DAKO Envision Detection System was used for detecting PD‐L1 staining. ABCG2, AHNAK2, BCL2, FZD1, and TP73 staining of the metastatic lymph node sample was performed using anti‐ABCG2 antibody (Clone B‐1; Santa Cruz Biotechnology, Dallas, TX, USA), anti‐AHNAK2 antibody (Clone AG11779; Proteintech, Rosemont, IL, USA), anti‐BCL2 antibody (Clone E17; Abcam), anti‐FZD1 antibody (Clone E‐7; Santa Cruz Biotechnology), and anti‐TP73 antibody (Clone EP436Y; Abcam) with detection carried out using DAB and Chromogen (DAKO, Glostrup, Denmark). Human placenta, skin, and tonsil tissues were used as positive controls for each primary antibody, placenta for ABCG2, tonsil for BCL2 and FZD1, skin for AHNAK2 and TP73.

## Results

**Figure 1:**
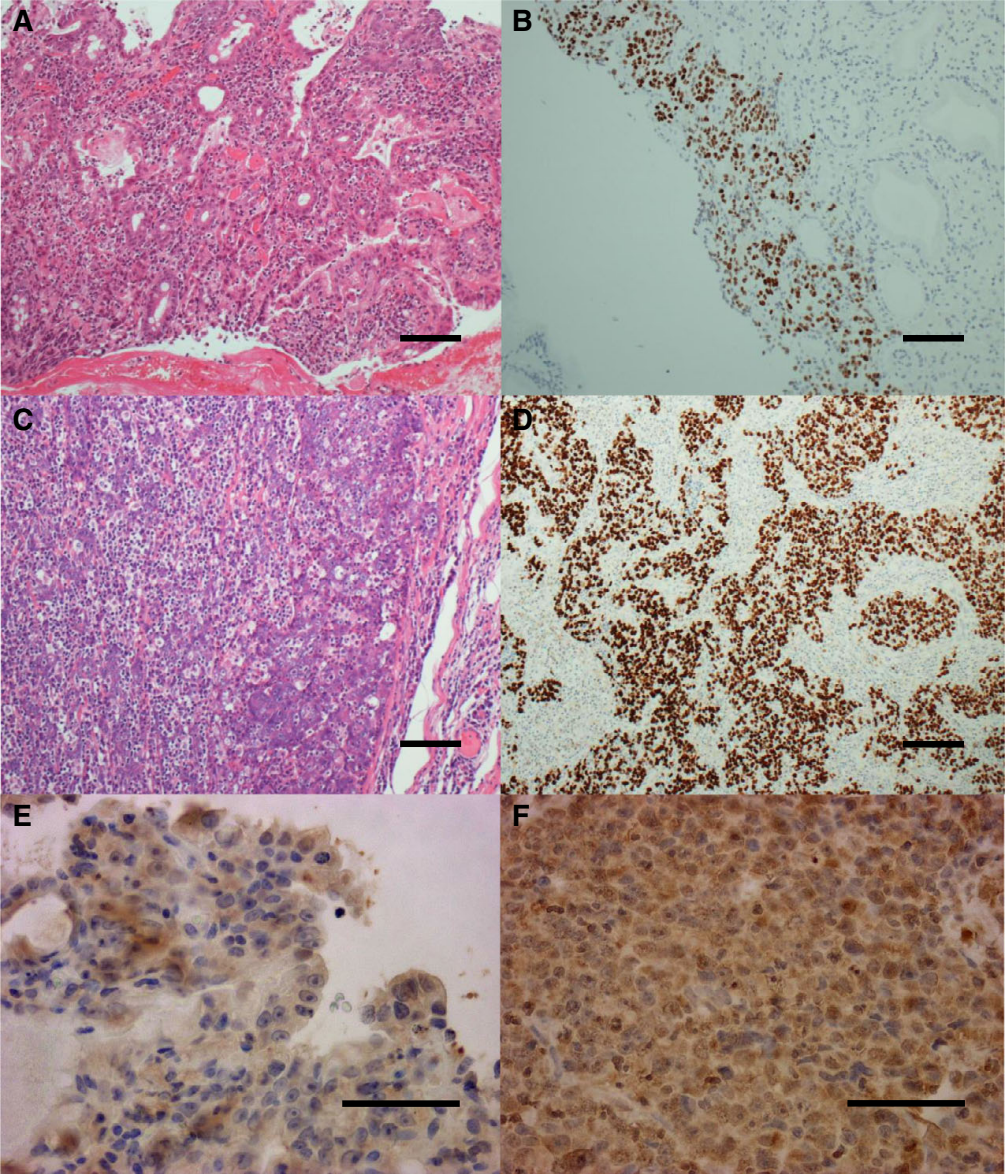
Histopathological examination of the biopsy from primary and metastatic lesion. (A, C) A metastatic lesion of lymph node and primary lesion were identified as a poorly to moderately differentiated adenocarcinoma (scale bar 100 μm); (B, D) EBER‐in situ hybridization of both lesions showed positive staining (scale bar 100 μm); (E, F) PD‐L1 staining was also positive in both lesions (scale bar 50 μm).

**Figure 2:**
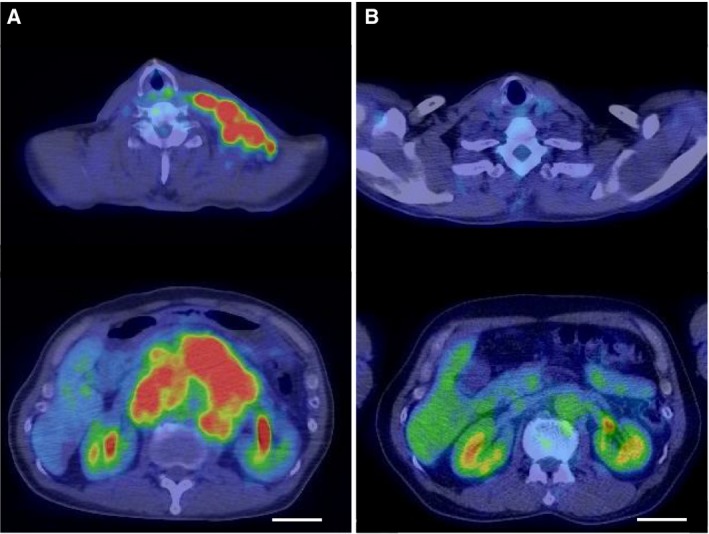
Radiological examination of FDG‐PET. (A) FDG‐PET showed abnormal uptake in regions with cervical and celiac multiple lymphadenopathy; (B) After 14 cycles of S‐1 plus cisplatin therapy, FDG‐PET showed no abnormal uptake (scale bar 5 cm).

**Figure 3:**
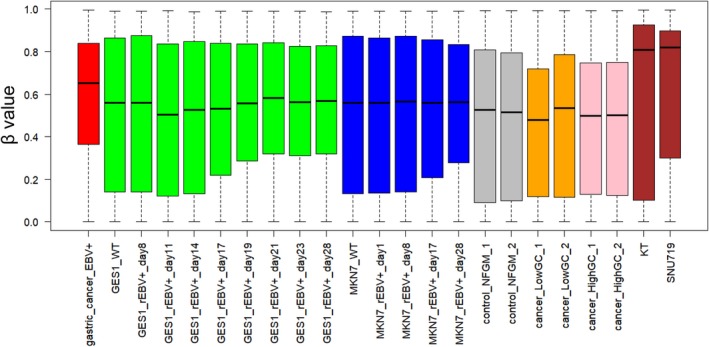
Boxplot of β values from ~ 450 000 CpG sites from each sample. Whiskers indicate minimum and maximum values, boxes indicate the first and third quartiles, and bold black lines indicate median values. Colors were assigned to each sample: red, the EBVGC case (gastric_cancer_EBV+); green, GES1 infected with EBV (GES1_rEBV_days) or without infection (GES1_WT); blue, MKN7 infected with EBV (MKN7_rEBV+_days) or without infection (MKN7_WT); gray, normal gastric mucosal cells (control_NFGM_1, control_NFGM_2); orange, hypomethylated clinical GC specimens (cancer_LowGC_1, cancer_LowGC_2); pink, hypermethylated clinical GC specimens (cancer_HighGC_1, cancer_HighGC_2); brown, EBVGC cell line and xenograft (SNU‐719, KT).

**Figure 4:**
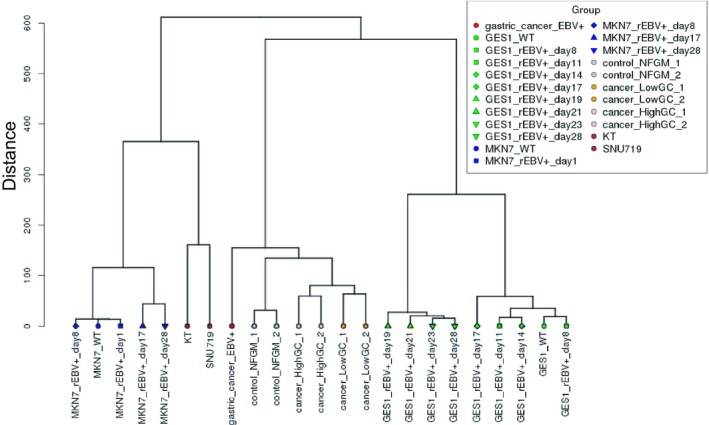
Hierarchical cluster analysis based on CpG methylation values.

**Figure 5:**
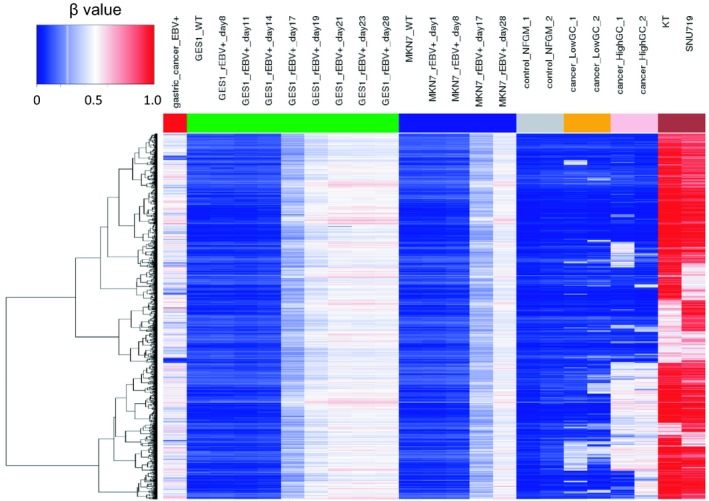
Heatmap analysis of probes meeting the extraction conditions. Extraction conditions were defined as: (a) Methylation negative in the control group (control_NFGM; β value < 0.2); (b) methylation positive in the EBVGC cell line and xenograft (SNU‐719, KT; β value > 0.4); (c) CpG regions which turned from methylation‐negative to methylation‐positive on day 28 following EBV infection in GES1 and MKN7 (GES1_WT β value < 0.2; GES1_rEBV _day28 β value > 0.4; MKN 7_WT β value < 0.2; MKN 7_rEBV_day28 β value > 0.4).

**Figure 6:**
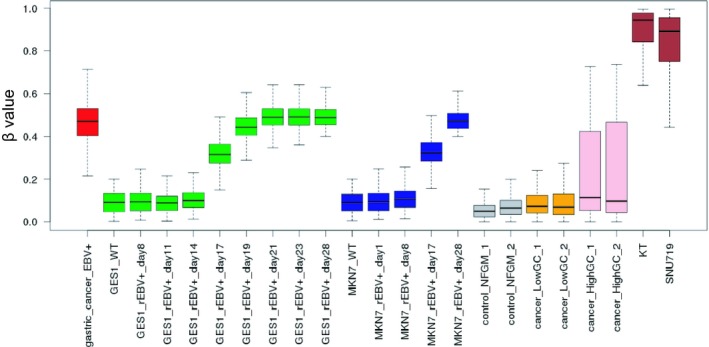
Boxplot of methylation level (β values) for probes meeting extraction conditions. Extraction conditions were the same as described in Fig. 5.

**Figure 7:**
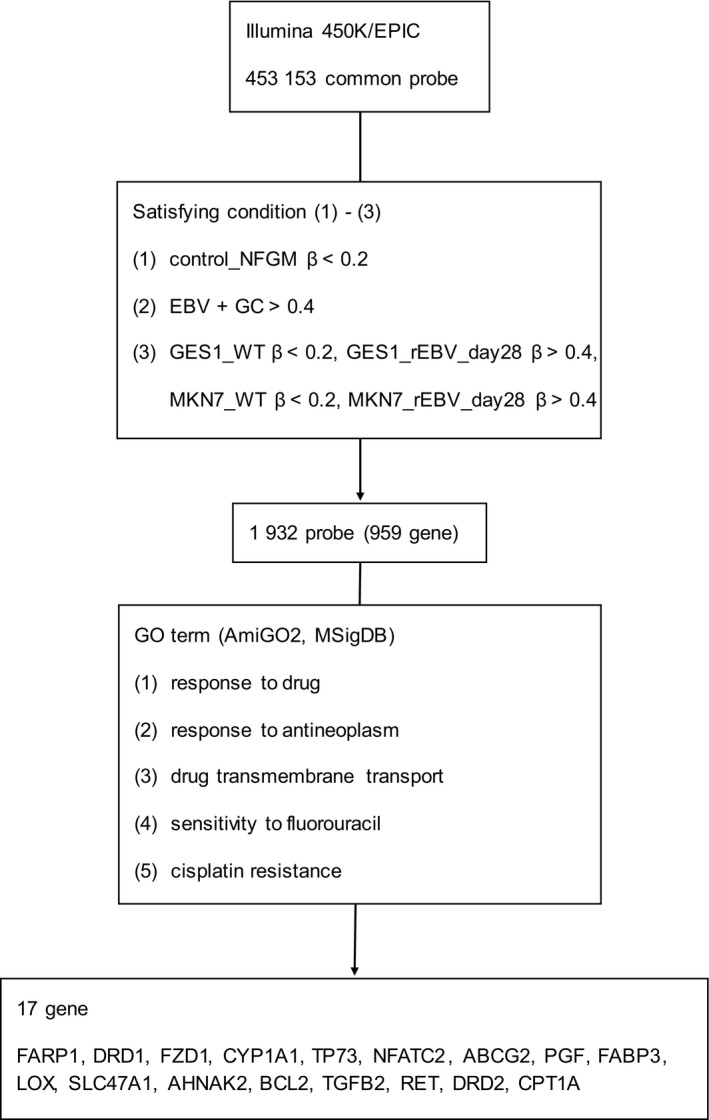
Flowchart of the extraction methodology for probes and genes.

**Figure 8:**
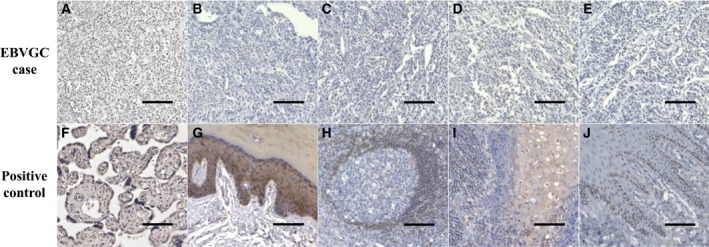
Gene expression in a metastatic lymph node lesion, measured using IHC in combination with hematoxylin. (A–E) High‐magnification view of metastatic lymph node lesions and (F–J) positive control (ABCG2, AHNAK2, BCL2, FZD1, and TP73 IHC, ×200, scale bar 100 μm) are shown.

**Figure 9:**
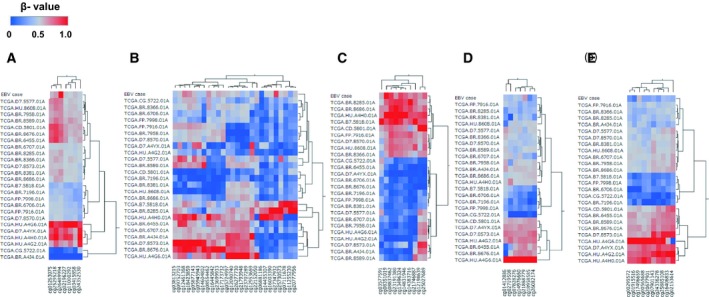
Heatmap analysis of chemoresistance‐associated gene methylation of EBVGC cases. The methylation patterns of each gene associated with chemoresistance, (A) ABCG2; (B) BCL2; (C) FZD1; (D) AHNAK2 and (E) TP73 were analyzed with heatmap and clustering analysis. The row indicates each case (EBV case; our case, rows which begin with TCGA; TCGA EBVGC cases), and the column indicates each probe corresponding to CpG sites in promotor region of each gene.

The results of histopathological examination of the primary lesion and metastatic lymph node were shown in Fig. 1. EBER‐in situ hybridization and PD‐L1 staining of both lesions were positive, consistent with the characteristics of EBVGC. The images of FDG‐PET before administration and after 14 cycles of S‐1 plus cisplatin are shown in Fig. 2. After 14 cycles of S‐1 plus cisplatin therapy, FDG‐PET showed no abnormal uptake. The methylation level (β value) of CpG sites within genomic DNA extracted from cervical lymph node metastatic tissue of the EBVGC case was analyzed using the EPIC platform. Methylation in the promoter regions of multiple tumor suppressor genes such as *APC*, *CDH1*, *THBS1*, *CDKN2A*, and *TP73* has been reported in previous studies of EBVGC 3, 4, 5. Methylation of these genes was similarly observed in the present EBVGC case using EPIC analysis. We annotated a gene ‘methylated’, if the β value was greater than 0.4. The median β value (±standard deviation, SD) of each gene was as follows: *APC*, 0.571 ± 0.289; *CDH1*, 0.180 ± 0.261; *THBS1*, 0.768 ± 0.259; *CDKN2A*, 0.495 ± 0.120; *TP73*, 0.549 ± 0.212. Two probes out of ten for *CDH1* showed β values higher than 0.4 (0.670 and 0.807). The methylation data from the EBVGC case were then analyzed in combination with those from a publicly accessible database 9. The β values of 453 152 genomic CpG sites in the present study were recalculated using the raw data of the respective reports and the distribution of these values is shown in the box plot in Fig. 3. Whiskers indicate minimum and maximum values, boxes indicate the first and third quartiles, and bold black lines indicate median values. Colors were assigned to each sample: red, the EBVGC case (gastric_cancer_EBV+); green, GES1 infected with EBV (GES1_rEBV_days) or without infection (GES1_WT); blue, MKN7 infected with EBV (MKN7_rEBV+_days) or without infection (MKN7_WT); gray, normal gastric mucosal cells (control_NFGM_1, control_NFGM_2); orange, hypomethylated clinical GC specimens (cancer_LowGC_1, cancer_LowGC_2); pink, hypermethylated clinical GC specimens (cancer_HighGC_1, cancer_HighGC_2); brown, EBVGC cell line and xenograft (SNU‐719, KT). The median β values (±SD) for the EBVGC case, cell lines infected with EBV (GES1, MKN7), and non‐EBVGC cases (cancer_LowGC, cancer_HighGC) were as follows: EBVGC, 0.651 ± 0.307; GES1_WT, 0.560 ± 0.339; MKN7_WT, 0.560 ± 0.345; cancer_LowGC_1, 0.477 ± 0.300; cancer_LowGC_2, 0.535 ± 0.321; cancer_HighGC_1, 0.499 ± 0.306; cancer_HighGC_2, 0.501 ± 0.308. The EBVGC sample showed significantly higher β values (*P* < 0.001) than the GC cell lines (GES1_WT, MKN7_WT) or the non‐EBVGC clinical samples in the Wilcoxon test. In the cell lines GES1 and MKN7, β values (median ± SD) were significantly higher at 28 days post‐EBV infection compared with EBV‐uninfected cells (GES1_rEBV+_day 28, 0.488 ± 0.294; MKN7_rEBV+_day 28, 0.470 ± 0.304) (Fig. 3). Hierarchical cluster analysis based on the β values of the 453 152 CpG sites is shown in Fig. 4. In this analysis, the EBVGC case was more closely clustered with the control group (control_NFGM) and the non‐EBVGC clinical samples (cancer_LowGC, cancer_HighGC) than with the GC cell lines. These data suggested that the global methylation pattern of the EBVGC cells more closely resembled that of normal human gastric mucosa rather than human GC cell lines. Since DNA methylation of the promoter region is involved in gene silencing, CpG islands in promoter regions that satisfy the following two criteria were selected to search the genes silenced by EBV infection: Category A, CpG islands not methylated in the control group (control_NFGM, β < 0.2) and CpG islands methylated in the EBVGC cell lines and xenografts (SNU‐719 and KT, β > 0.4); Category B, CpG regions which turned from methylation‐negative to methylation‐positive on day 28 following EBV infection in GES1 and MKN7 (GES1_WT, β < 0.2; GES1_rEBV _day 28, β > 0.4; MKN 7_WT, β < 0.2; MKN 7_rEBV_day 28, β > 0.4). One thousand nine hundred thirty‐two CpG islands, which represent 959 genes, matched both categories. Heatmap of the EBVGC case and the other samples in terms of these selected CpG regions was then generated (Fig. 5), and the DNA methylation rates shown by box plot (Fig. 6). The DNA methylation rate of the EBVGC case was significantly higher than that of the control group and non‐EBVGC cases (*P* < 0.001). EBV‐infected cell lines (GES1, MKN7) showed an increase in the methylation rate in a time‐dependent manner, with the day 28 methylation rates being almost equivalent to that of the EBVGC case. The EBVGC cell lines and xenografts (SNU‐719 and KT) showed particularly high methylation rates. In contrast, the methylation rate of the extracted CpG regions was found to be low in normal gastric mucosal tissue. Since the EBVGC case exhibited prominent sensitivity to chemotherapy consisting of fluoropyrimidine and platinum, we examined the DNA methylation status of selected genes for a potential correlation with drug sensitivity and resistance. Among the 959 genes identified above, a subset of genes related to drug susceptibility or resistance was extracted using the gene ontology search software of the MSigDB and AmiGO2 sites 12, 13, 14, 15. The keywords used to identify these genes were 5‐fluorouracil (5‐FU) sensitivity, cisplatin resistance, drug susceptibility, and drug transporter. Finally, 17 genes were identified (*AHNAK2, FARP1, BCL2, DRD1, FZD1, CYP1A1, TP73, NFATC2, TGFB2, ABCG2, RET, DRD2, CPT1A, PGF, FABP3, LOX, SLC47A1*) as the genes related to drug susceptibility or resistance silenced by EBV infection. The algorithm for selecting the CpG regions and genes is shown in Fig. 7. Among these 17 genes, all except for *FARP1* were positive for methylation in the corresponding promoter regions in the EBVGC case. Furthermore, the genes involved in 5‐FU and cisplatin resistance were searched on PubMed, which found five of the above genes (*ABCG2, AHNAK2, BCL2, FZD1, TP73*) that have a reported association with cisplatin resistance. From these five genes, an immunohistochemical assessment of ABCG2, AHNAK2, BCL2, FZD1, and TP73 expression was performed using the metastatic lymph node FFPE sample (Fig. 8). In contrast to the positive control sample, no staining of ABCG2, AHNAK2, BCL2, FZD1 and TP73 was observed in the metastatic EBVGC lesion. These results suggested that DNA methylation in the promoter region might possibly have caused gene silencing. We also compared the methylation patterns of CpG sites in promoter region of extracted these genes between our EBVGC case and TCGA cases with heatmap and clustering analysis. The heat maps of methylation pattern are shown in Fig. 9. The row indicates each case (EBV case; our case, rows which begin with TCGA; TCGA EBVGC cases), and the column indicates each probe corresponding to CpG sites in promotor region of each gene. Methylation patterns are classified into several groups by clustering analysis, which suggesting that DNA methylation profiles of genes associated with chemoresistance are different between each EBVGC case.

## Discussion

S‐1 plus cisplatin therapy has been shown to significantly prolong overall survival and has been one of the standard treatments for HER2‐negative GC 16. However, the reported complete response rate of treatment groups is extremely low (~ 1%). Even with triplet therapy consisting of docetaxel, cisplatin and 5‐FU for advanced GC, the complete response rate has been reported to be as low as 2% 17. In this context, the present EBVGC case can therefore be considered as being highly sensitive to chemotherapy. EBVGC is one of the molecular subtypes of GC reported by the TCGA and prognosis is better than that of non‐EBVGC at the same stage 6. However, there has been no report of the survival benefit of chemotherapies for advanced EBVGCs. In addition, since the proportion of EBVGC cases in the pivotal clinical trials has not been examined, the relative efficacy of chemotherapy in this group remains undetermined. EBVGC was reported to be resistant to chemotherapy in previous *in vitro* studies 18, 19. On the other hand, *in vitro* and *in vivo* studies have reported that certain anticancer agents, such as gemcitabine, induced a therapeutic effect by modulating EBV‐infected cells from a latent state to lytic infection 20, 21. Since EBVGC is a relatively rare subtype, investigating a correlation of the biological features of EBVGC with clinical data is thought to be necessary. Although hypermethylation of DNA at promoter regions, a putative mechanism contributing carcinogenesis, is induced by EBV infection in gastric epithelial cells 3, information regarding specifically methylated genes in clinical specimens of EBVGC is limited. In the present study, methylation of the promoter regions of multiple tumor suppressor genes, such as *APC*, *CDH1*, *THBS1*, *CDKN2A*, and *TP73*, was observed in the EBVGC case. Since this case was highly sensitive to chemotherapy, we searched gene‐sets related to drug sensitivity and resistance using gene ontology terms in addition to EBVGC‐specific genes that have already been reported to be methylated in promoter regions. As a result from analysis on the present EBVGC case and EBVGC cell lines from a previously reported dataset, we found DNA methylation at the promoter regions of the genes *ABCG2, TP73, AHNAK2, BCL2,* and *FZD1* that are reported to be associated with drug resistance 22, 23, 24, 25, 26. This suggested that silencing of drug resistance genes might contribute to the high sensitivity to chemotherapy in the present case. Indeed, negative expression of the ABCG2, AHNAK2, BCL2, FZD1, and TP73 protein in the EBVGC metastatic site was confirmed by IHC. We also compared the methylation of these genes between our EBVGC case and TCGA cases and different methylation patterns were observed indicating different chemosensitivity of EBVGC case; however, clinical data of chemotherapy, such as chemosensitivity or chemoresistance, were not included in the database. Therefore, it is thought to be difficult to pursue the association between chemosensitivity and methylation pattern from public data because of the lack of clinical data. The MSI subtype, another molecular subtype of GC, is known to be associated with DNA methylation of *MLH1* which is not methylated in EBVGC 4. Among the common probes between the 450K and EPIC platforms, there are 32 probes for *MLH1*. Twenty‐six probes out of 32 target the promoter region and the remaining six probes target the gene body. Neither of normal gastric mucosal cells (control_NFGM_1 and control_NFGM_2) have no methylation at promoter region of MLH1 and there was no significant difference between normal mucosa and EBVG case (Wilcoxon test, *P* = 0.422 and 0.198, respectively), and the methylation pattern was similar to the heatmap (figure not shown). We conclude that there was no significant difference between the EBVGC case and the NFGM cases in terms of the methylation of the *MLH1* promotor. In contrast, the *MLH1* promotor was more methylated in the cancer_HighGC_2 case than the EBVGC or cancer_HighGC_1 cases (Wilcoxon, *P* < 0.005), which suggests the phenotype of an MSI subtype for the former case (cancer_HighGC_2), although mutation of genomic DNA was not analyzed in the dataset. The median β values (±SD) for each case in regard to the *MLH1* promotor region were as follows: EBVGC, 0.044 ± 0.197; control_NFGM_1, 0.059 ± 0.070; control_NFGM_2, 0.054 ± 0.095; cancer_HighGC_1, 0.081 ± 0.092; cancer_HighGC_2, 0.438 ± 0.061. There is no public database of comprehensive DNA methylation analysis by EPIC for EBVGC. Therefore, the methylation data for the present case were compared with public data from various normal gastric mucosa and GC cell types derived using the 450K platform. In the upgrade from the 450K analytical platform to EPIC, 381 318 new probes covering the transcriptional regulatory region were added. The total number of EPIC probes is ~ 850 000, while the number of probes on the 450K is ~ 480 000, and most of the 450K probes have been passed over to EPIC 27. Thus, a significant amount of data can be compared between these two platforms. However, among the methylation data analyzed by EPIC, ~ 400 000 newly added CpG regions are excluded from the comparative analysis. Several studies have reported that genetic alterations in enhancer regions caused by EBV infection are associated with carcinogenesis 28. Therefore, further analyses of the methylation of enhancer regions might help to elucidate the mechanisms of carcinogenesis in EBVGC. Interestingly, the DNA methylation rate of the EBVGC case was observed to be higher than that of the control group and non‐EBVGC cases and was almost equivalent to experimentally EBV‐infected cell lines (GES1, MKN7) on day 28. In the hierarchical clustering analysis, the EBVGC case was classified into the cluster close to the normal gastric mucosa and non‐EBVGC cases, and distant from the GC cell lines infected by EBV. Taken together, our findings indicate that the promotor methylation status of EBVGC is similar to other clinical GC samples or GC cells shortly after EBV infection and suggest that the methylation status of a relatively limited number of CpG regions contributes to the key biological features of this phenotype. This study is the first comprehensive analysis of DNA methylation in an EBVGC case exhibiting high sensitivity to chemotherapy using the Infinium MethylationEPIC BeadChip Kit, which is Illumina's latest methylome analysis platform. In the TCGA database, the previous Infinium Human Methylation450K BeadChip or Infinium Human Methylation27 BeadChip Kits have instead been used for analyses 4. Accumulation of DNA methylation data for EBVGC is important to increase our understanding of both of the mechanisms of tumorigenesis and the most effective therapeutic strategies.

## Conflict of interest

EB and KT received honoraria from Taiho Pharma. KA received research funding from Taiho Pharma. The remaining authors declare no conflict of interest.

## Author contributions

HO wrote the main manuscript text and prepared figures with support from EB, MI, and TI, CO and TS contributed to statistical analysis and interpretation of data and assisted in the preparation of the manuscript. All other authors have contributed to data collection and interpretation and critically reviewed the manuscript.

## Data accessibility

The methylation data have been deposited in NCBI's Gene Expression Omnibus (Edgar *et al*., 2002) and are accessible through GEO Series accession number http://www.ncbi.nlm.nih.gov/geo/query/acc.cgi?acc=GSE132406.
